# Hedgehog Interacting Protein Promotes Fibrosis and Apoptosis in Glomerular Endothelial Cells in Murine Diabetes

**DOI:** 10.1038/s41598-018-24220-6

**Published:** 2018-04-13

**Authors:** Xin-Ping Zhao, Shiao-Ying Chang, Min-Chun Liao, Chao-Sheng Lo, Isabelle Chenier, Hongyu Luo, Jean-Louis Chiasson, Julie R. Ingelfinger, John S. D. Chan, Shao-Ling Zhang

**Affiliations:** 1 Université de Montréal Department of Medicine Centre de recherche du Centre hospitalier de l’Université de Montréal (CRCHUM) Tour Viger, 900 rue Saint-Denis, Montréal, QC H2X 0A9 Canada; 2 Harvard Medical School Pediatric Nephrology, Unit Massachusetts General Hospital 55 Fruit Street, Boston, MA 02114-3117 USA

## Abstract

We investigated whether renal hedgehog interacting protein (Hhip) expression contributes to the progression of diabetic nephropathy (DN) and studied its related mechanism(s) *in vivo* and *in vitro*. Here, we show that Hhip expression is highly elevated in glomerular endothelial cells of adult type 1 diabetic (T1D) Akita and T2D *db/db* mouse kidneys as compared to non-diabetic control littermates. Hyperglycemia enhances reactive oxygen species (ROS) generation via NADPH oxidase 4 (Nox4) activation and stimulates renal Hhip gene expression, and that elevated renal Hhip gene expression subsequently activates the TGFβ1- Smad2/3 cascade and promotes endothelial to mesenchymal transition associated with endothelial cell fibrosis/apoptosis *in vivo* and *in vitro*. Furthermore, kidneys of low-dose streptozotocin-induced diabetic heterozygous Hhip deficient (Hhip^+/−^) mice displayed a normal albumin/creatinine ratio with fewer features of DN (glomerulosclerosis/fibrosis and podocyte apoptosis/loss) and less evidence of renal compensation (glomerular hypertrophy and hyperfiltration) as compared to diabetic wild type controls (Hhip^+/+^). Thus, our studies demonstrated that renal Hhip expression is associated with nephropathy development in diabetes and that hyperglycemia-induced renal Hhip expression may mediate glomerular endothelial fibrosis and apoptosis in diabetes, a novel finding.

## Introduction

In patients with diabetes, endothelial injury leads to multiple macro- and microvascular complications, including diabetic nephropathy (DN). DN accounts for 50% of all end-stage renal disease (ESRD) cases in Canada and the USA^1–3^, and the incidence of DN, a progressive kidney disease, is increasing world wide^4^. In DN, the glomerular filtration barrier, which consists of an inner fenestrated glomerular endothelial cells (GECs) layer, a glomerular basement membrane (GBM) and an outer layer of podocytes with interdigitated foot processes that enwrap the glomerular capillaries, is injured. GEC injury (reduced fenestrated capacity/ability), GBM thickening, and podocyte foot process effacement/detachment are hallmarks of early renal injury in DN^1–3^. Although current treatments such as anti-hyperglycemic agents, statins, renin-angiotensin system (RAS) blockers and other anti-hypertensive agents can slow the progression of DN, such agents have failed to prevent the development of ESRD. Therefore, the identification of new molecules that might be useful to develop targeted preventive therapies for patients at risk for developing DN would be important.

Hedgehog interacting protein (Hhip), a signaling molecule in the hedgehog (Hh) pathway, was originally discovered as a putative antagonist of all 3 secreted Hh ligands, i.e., Sonic (Shh), Indian (Ihh), and Desert (Dhh)^5–10^. Hhip encodes a protein of 700 amino acids attached to the cell membrane via a glycosylphosphatidylinositol (GPI) anchor and is abundantly expressed in vascular endothelial cells-rich tissues, including the kidney^11^. Hhip regulates cell function via either canonical- or non-canonical hedgehog pathways^5–10,12,13^. Under certain conditions, cells express Hhip, which then acts cell-autonomously (intrinsically, in the same and/or adjacent cells – in an “autocrine manner”) and non-cell autonomously (acting in other cells – in a “paracrine manner”) to regulate cell and/or organ functions^5–10,12,13^. Hhip function is important during organogenesis as interruption of *Hhip* gene expression results in developmental anomalies that include skeletal, lung and pancreatic malformations^7,12,14,15^. In contrast, *Hhip* expression is quiescent after birth, but abnormal *Hhip* expression has been linked to several human diseases, such as pancreatitis^16^, chronic obstructive pulmonary disease^13,17,18^, and various tumors^11,19^. However, the pathophysiological role of Hhip in the kidney is poorly understood.

We recently discovered that *Hhip* gene expression is differentially up-regulated in the kidneys of the offspring in our murine model of maternal diabetes, impairing nephrogenesis^20^. Using cultured metanephric mesenchymal cells^21^, we demonstrated that high glucose (25 mM D-Glucose) specifically stimulated *Hhip* gene expression in a time- and dose-dependent manner. The hyperglycemic milieu delayed or disrupted the usual gradient Hhip-Shh expression pattern, and the elevated *Hhip* gene expression could be reversed by insulin^20^, suggesting that *Hhip* gene expression could be altered by hyperglycemia. In the present study, we hypothesized that hyperglycemia regulates *Hhip* gene expression and that elevated renal *Hhip* gene expression contributes to DN development and progression.

Here we examined the role of renal Hhip expression in murine models of diabetes mellitus—T1DM (in Akita mice^22,23^ and in low-dose streptozotocin (STZ) (LDSTZ)-induced diabetic heterozygous Hhip (Hhip^+/−^) mice^24,25^) and T2DM (*db/db* mice)^24,26,27^. We determined the mechanisms of hyperglycemia-induced renal *Hhip* gene expression that result in apoptosis of GECs and endothelial to mesenchymal transition (EndoMT)-related renal fibrosis.

## Results

### Hyperglycemia–Induced Renal *Hhip* Gene Expression

**Figure 1:**
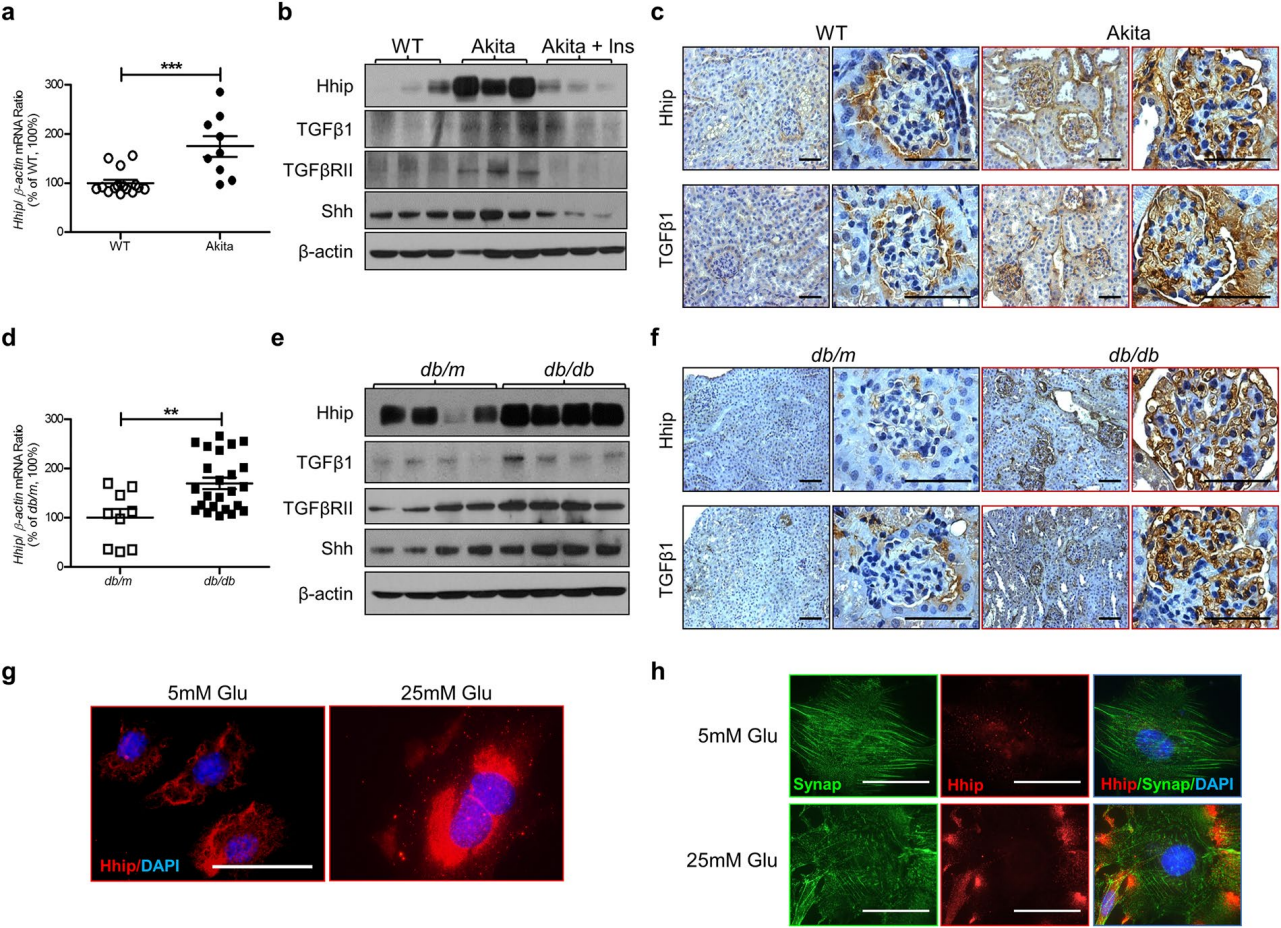
Hyperglycemia-induced renal Hhip expression *in vivo* (Akita (**a**–**c**) and *db/db* mice (**d**–**f**) at the age of 20 weeks) and *in vitro* (mECs (**g**) and mPODs (**h**)). (**a**,**d**) qPCR of Hhip mRNA expression in renal cortex. Hhip mRNA expression were normalized by their corresponding β-actin mRNA. (**b**,**e**) WB analysis of Hhip, Shh, TGFβ1 and TGFβ1RII in renal cortex. **P ≤ 0.01; ***P ≤ 0.001 vs. WT or *db/m*; Values represent the mean ± SEM. (**c**,**f**) Hhip- and TGFβ1-IHC in the kidneys (scale bar, 50 µm). (**g**–**h**) IF staining in mECs (**g**) and mPODs (**h**) (scale bar, 50 µm).

As compared to controls (non-Akita littermates (Fig. 1a–c) and *db/m* mice (Fig. 1d–f)), renal Hhip mRNA and protein expression were significantly increased in the renal cortex of both Akita (Fig. 1a–c) and *db/db* (Fig. 1d–f) mice at the age of 20 weeks. Western blot (WB) revealed that enhanced TGFβ1, TGFβ receptor II (TGFβRII) and Shh protein expression were also apparent in both diabetic models (Figs 1b, S1a and e, S1b). The increased Hhip, TGFβ1, TGFβRII and Shh protein expression in the renal cortex of Akita mice was normalized with insulin implants in the animals (Figs 1b and S1a). The heightened renal Hhip expression in both Akita and *db/db* mice was subsequently confirmed by immunohistochemistry (IHC) staining (Fig. 1c, 1f, respectively); TGFβ1 had a similar IHC expression pattern in Akita and *db/db* mice kidneys (Fig. 1c, 1f, respectively). Next, we validated our *in vivo* Hhip expression pattern by using 2 cell lines including murine SVEC4-10 endothelial cells (mECs) (ATCC, CRL-2181) (Fig. 1g) and immortalized mouse podocyte cells (mPODs)^28–30^ (Fig. 1h). It is apparent that high glucose (25 mM D-Glucose) increases Hhip protein expression in both mECs and mPODs, while it inhibits synaptopodin protein expression in mPODs (Fig. 1h).

**Figure 2:**
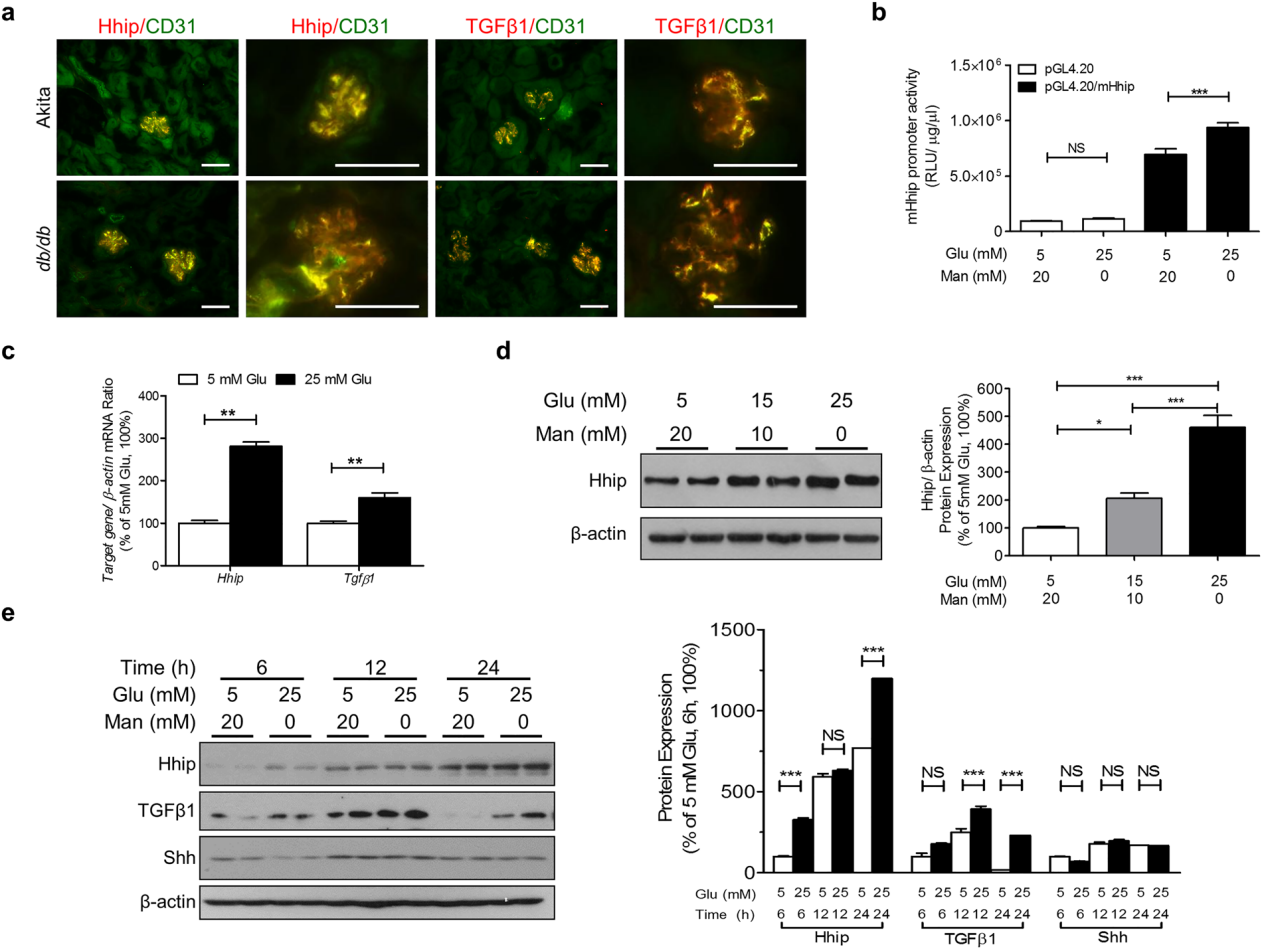
*Hhip* gene expression in GECs *in vivo* (**a**) and in mECs *in vitro* (**b**–**e**). (**a**) Hhip- and TGFβ1- co-localization IF-staining with CD31 in the kidney of Akita and *db/db* mice at the age of 20 weeks (scale bar, 50 µm); (**b**) pGL4.2/mHhip promoter activity analyzed by luciferase assay. ***P ≤ 0.001; NS, non-significant; Values represent the mean ± SEM. (**c**) qPCR of Hhip mRNA. Hhip mRNA expression were normalized by their corresponding β-actin mRNA; (**d**,**e**) WB analysis in glucose dose- (**d**) and time- (**e**) dependent manner, *P ≤ 0.05; **P ≤ 0.01; ***P ≤ 0.001 vs. mECs cultured in 5 mM glucose (100%); Values represent the mean ± SEM.

In line with the Hhip expression reported in endothelial cells^11^, our co-localization experiments (Fig. 2a, immunofluorescence (IF)) showed that hyperglycemia-induced Hhip and TGFβ1-IF expression was predominantly found in GECs (revealed by CD31 co-localization), suggesting that GECs might be the potential source of elevated Hhip expression in diabetic glomeruli. Focusing on mECs (Fig. 2b–e), our data revealed that high glucose stimulates pGL4.20/mHhip promoter (N-1542/N+9, NC_000074.6) activity (Fig. 2b), Hhip mRNA (Fig. 2c) and protein (Fig. 2d–e) expression in a dose-dependent and/or time-dependent manner. Furthermore, the stimulatory effect of high glucose on TGFβ1 mRNA (Fig. 2c) and protein expression (Fig. 2e) was similar to Hhip expression in mECs, while high glucose had no impact on Shh protein expression within 24 hours (Fig. 2e).

### Oxidative Stress and Hhip Expression

**Figure 3:**
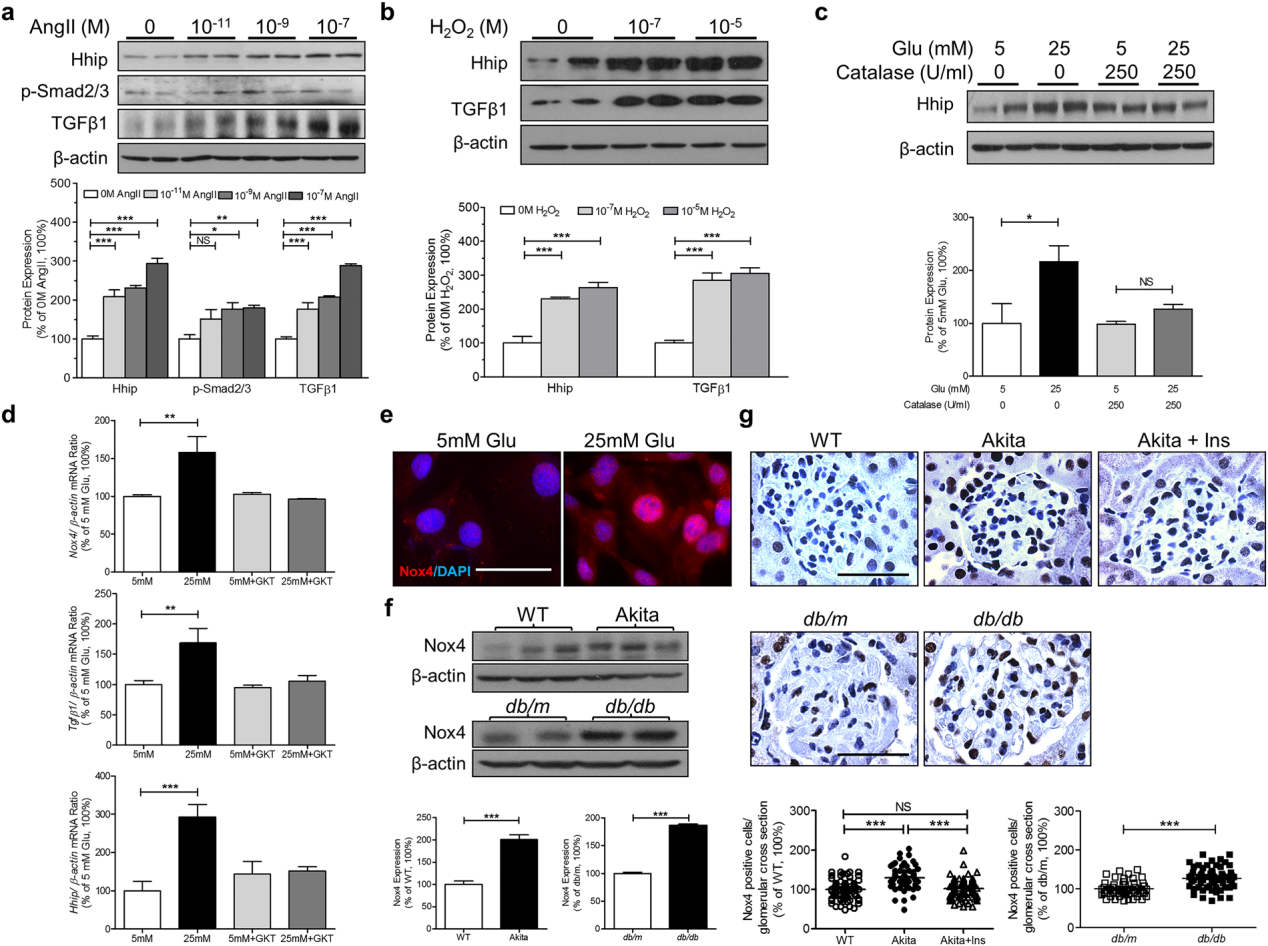
The interaction of Hhip and Nox4 *in vitro* and *in vivo*. (**a**–**c**) WB in mECs. (**a**) Ang II dose-dependent manner. (**b**) H_2_O_2_ dose-dependent manner. (**c**) High glucose ± Catalase (250U/ml) on Hhip protein expression. *P ≤ 0.05; **P ≤ 0.01; ***P ≤ 0.001; NS, non-significant vs. mECs cultured in 5 mM glucose (100%); Values represent the mean ± SEM. (**d**) qPCR of Nox4, TGFβ1 and Hhip- mRNA expression in mECs. mRNA of genes were normalized by their corresponding β-actin mRNA. **P ≤ 0.01; ***P ≤ 0.001; NS, non-significant vs. mECs cultured in 5 mM glucose (100%); Values represent the mean ± SEM. (**e**) Nox4-IF staining in mECs (scale bar, 50 µm). (**f**) WB of Nox4 protein expression in renal cortex of Akita and *db/db* mice at the age of 20 weeks. ***P ≤ 0.001 vs. WT or *db/m*; Values represent the mean ± SEM. (**g**) Nox4-IHC in the kidneys of Akita and *db/db* mice at the age of 20 weeks (scale bar, 50 µm). Semi-quantification of Nox4 positive stained cells per glomerulus. ***P ≤ 0.001; NS, non-significant.

Next, we investigated the effect of angiotensin II (Ang II) and hydrogen peroxide (H_2_O_2_) on Hhip protein expression. We observed that Ang II (Fig. 3a) and H_2_O_2_ (Fig. 3b) increased the expression of Hhip protein and activated the TGFβ1-Smad2/3 cascade in a dose-dependent manner. Catalase (250 U/ml) attenuated the stimulatory effect of high glucose on Hhip protein expression in mECs (Fig. 3c).

NADPH oxidase 4 (Nox4) is the major H_2_O_2_-generating enzyme expressed in endothelial cells^31^. The addition of 10 µM of GKT137831 (a dual inhibitor of both Nox1 and Nox4) completely abolished the high glucose effect on *Nox4*, *Hhip* and *TGFβ1*-mRNA expression in mECs (Fig. 3d). The increased Nox4 protein expression in mECs by high glucose was further confirmed by IF staining (Fig. 3e). Similarly, as compared to controls (non-Akita littermates and *db/m* mice), Nox4 protein expression was highly elevated in the renal cortex of both Akita and *db/db* mice at the age of 20 weeks as analyzed by WB (Fig. 3f) and it was profoundly increased in the glomeruli as revealed by Nox4-IHC staining (Fig. 3g).

**Figure 4:**
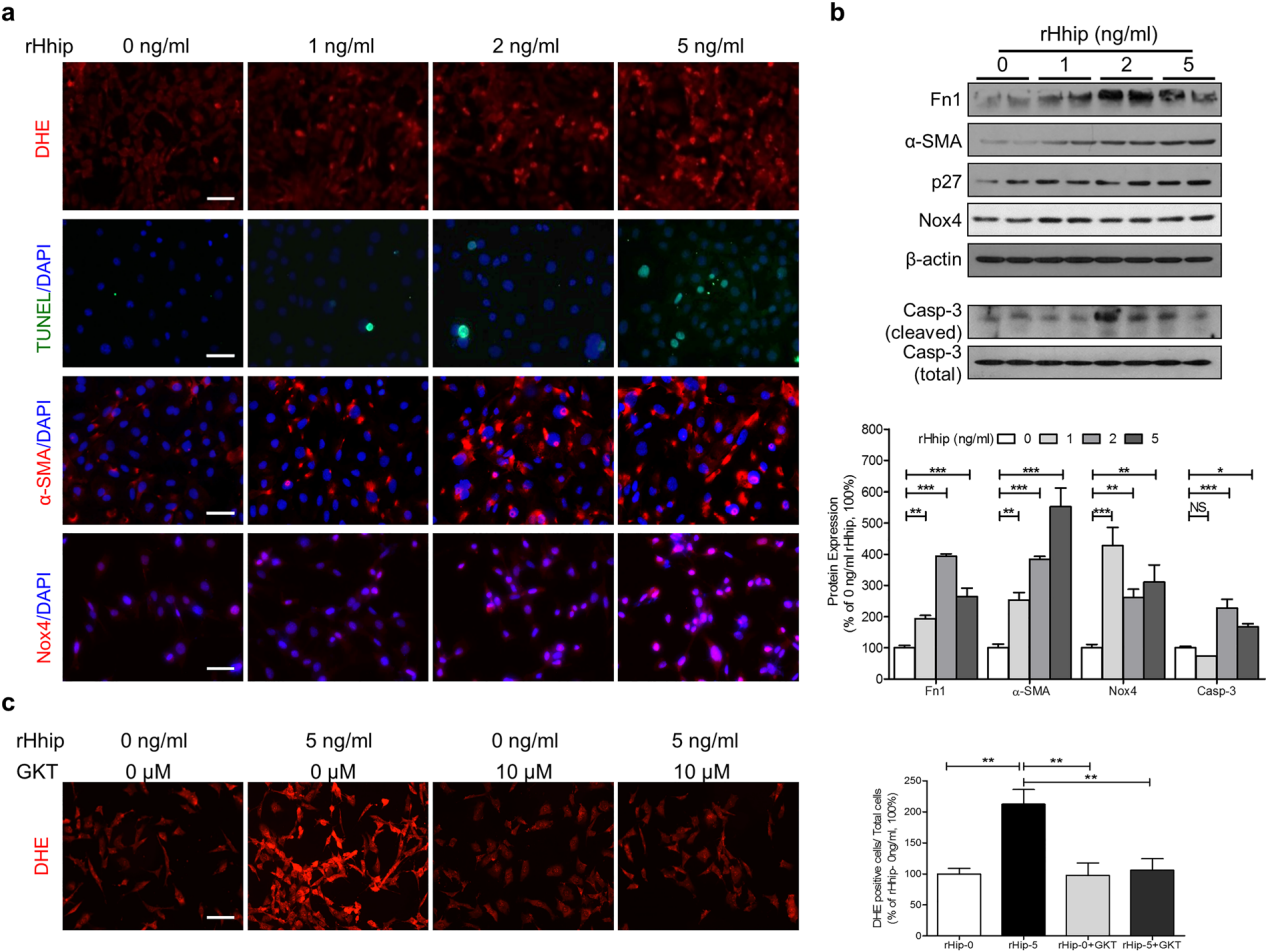
rHhip effect in mECs. (**a**) rHhip dose-dependent effect analyzed by DHE staining, TUNEL, α-SMA- and Nox4-IF staining (scale bar, 50 µm). (**b**) rHhip dose-dependent effect on a variety of proteins expression analyzed by WB. *P ≤ 0.05; **P ≤ 0.01; ***P ≤ 0.001; NS, non-significant vs. mECs cultured in 5 mM glucose (100%) without rHhip (0 ng/ml); Values represent the mean ± SEM. (**c**) The inhibitory effect of GKT137831 (10 µM) on DHE staining with or without rHhip (5 ng/ml) (scale bar, 50 µm). **P ≤ 0.01; ***P ≤ 0.001; NS, non-significant vs. mECs cultured in 5 mM glucose (100%) without rHhip (0 ng/ml).

Recombinant Hhip (rHhip) dose-dependently enhanced the number of DHE-positive cells and apoptotic cells (terminal deoxynucleotidyl transferase dUTP nick end labeling (TUNEL) assay), and increased α-smooth muscle actin (α-SMA) and Nox4 IF-staining (Fig. 4a), as well as the expression of several factors associated with fibrosis and apoptosis in mECs as shown by changes in the expression of fibronectin (Fn1), α-SMA, Shh, p27, Nox4 and cleaved caspase-3 (Fig. 4b). The stimulatory effect of rHhip on dihydroethidium (DHE) staining was completely reversed by GKT137831 (10 µM) (Fig. 4c).

### The Interaction of Hhip and TGFβ1 *in vitro*

**Figure 5:**
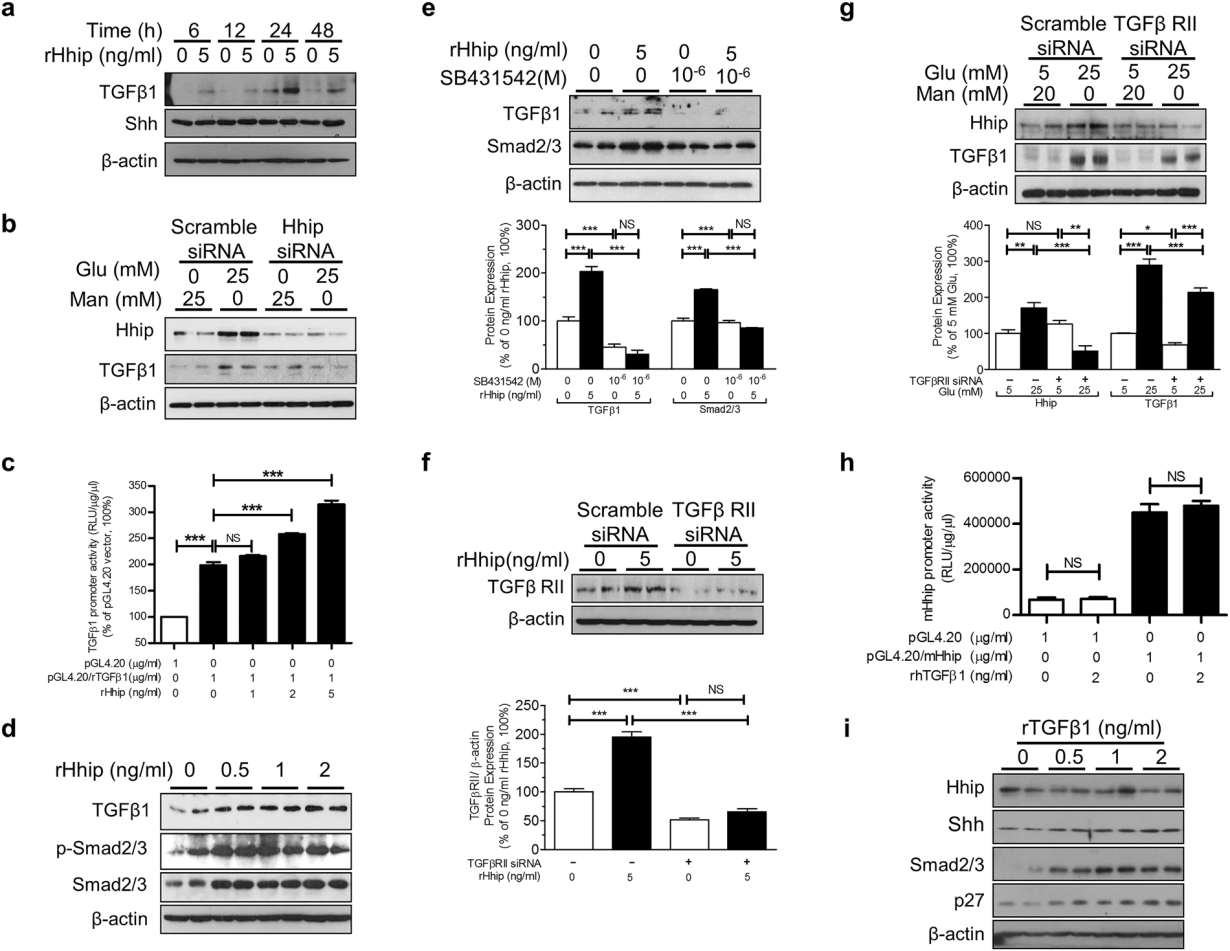
The interaction of Hhip and TGFβ1 signaling in mECs *in vitro*. (**a**) rHhip treatment in a time-course on Shh and TGFβ1 protein expression analyzed by WB. ***P ≤ 0.001 vs. mECs cultured in rHhip (0 ng/ml) at 6 h (100%), NS, non-significant; Values represent the mean ± SEM. (**b**) WB analysis of Hhip siRNA treatment. (**c**) rHhip effect on pGL4.2/rTGFβ1 promoter activity analyzed by luciferase assay. ***P ≤ 0.001 vs. mECs transfected with pGL4.20 (1 µg/ml) (100%); Values represent the mean ± SEM. (**d**) rHhip effects on TGFβ1 and phosphorylation of Smad 2/3 expression analyzed by WB. (**e**) WB analysis of rHhip ± SB431532 treatment. ***P ≤ 0.001 vs. mECs cultured in rHhip (0 ng/ml) (100%), NS, non-significant; Values represent the mean ± SEM. (**f**) WB analysis of rHhip ± TGFβII siRNA treatment. ***P ≤ 0.001 vs. mECs cultured in rHhip (0 ng/ml) (100%), NS, non-significant; Values represent the mean ± SEM. (**g**) WB analysis of TGFβII siRNA treatment. *P ≤ 0.05; **P ≤ 0.01; ***P ≤ 0.001; NS, non-significant vs. mECs cultured in 5 mM glucose without TGFβRII siRNA (100%); Values represent the mean ± SEM. (**h**) rTGFβ1 effect on pGL4.2/mHhip promoter activity analyzed by luciferase assay. NS, non-significant. (**i**) rTGFβ1 dose-dependent effect on protein expression analyzed by WB.

rHhip directly activated TGFβ1 protein expression in a time-dependent manner without influencing Shh protein expression (Figs 5a and S2a) in mECs. Transient transfection of Hhip siRNA attenuated the high glucose-stimulatory effects on both Hhip and TGFβ1 protein expression in mECs (Figs 5b and S2b). Also, rHhip dose-dependently stimulated pGL4.20/TGFβ1 promoter activity (Fig. 5c) and activated TGFβ1-Smad2/3 cascades (Figs 5d and S2c) in mECs. Furthermore, blocking TGFβ1 receptors (TGFβ1RI/RII), either by SB431532 (an inhibitor of TGFβ1RI) (Fig. 5e) or TGFβ1RII siRNA (Fig. 5f–g), abolished the stimulatory effect of rHhip (Fig. 5e–f) and high glucose (Fig. 5g) on TGFβ1 protein expression. In contrast, administration of recombinant TGFβ1 (2 ng/ml) had no impact on either pGL4.20/mHhip promoter (N-1016/+143, NM_021578.2) activity (Fig. 5h) or Hhip protein expression (Fig. 5i) in mECs.

### Low Dose Streptozotocin (LDSTZ)-Induced Diabetes in Heterozygous Hhip^+/−^ Mice

**Figure 6:**
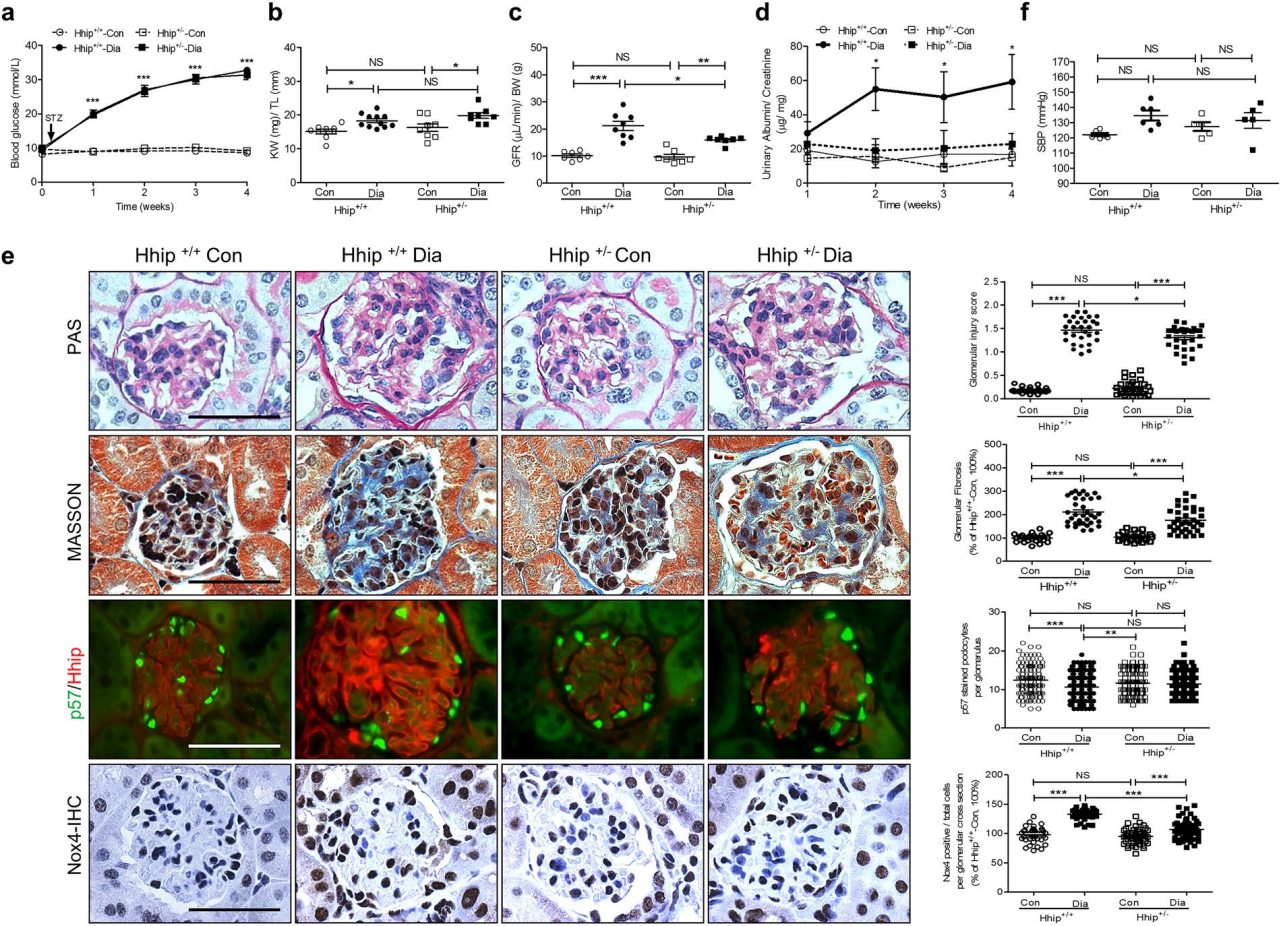
4 weeks of LDSTZ-induced diabetic model in male Hhip^+/+^ and Hhip^+/−^ mice from the age of 12 to 16 weeks. (**a**) Glycemia measurement. (**b**) Ratio of KW/TL. (**c**) GFR measurement. (**d**) Urinary ACR measurement. (**e**) Renal morphology. PAS staining (scale bar, 50 µm) with semi-quantification of glomerulosclerosis injury score. Grade 0, normal glomeruli; Grade 1, presence of mesangial expansion/thickening of the basement membrane; Grade 2, mild/moderate segmental hyalinosis/sclerosis involving less than 50% total glomerular area; Masson staining (scale bar, 50 µm) with semi-quantification of glomerular fibrosis; p57/Hhip co-IF staining with semi-quantification of p57 positive stained cells per glomerulus. Nox4-IHC staining with semi-quantification of Nox4 positive stained cells per glomerulus. (**f**) SBP (mmHg) measurement. NS, non-significant vs. Hhip^+/+^-Con; Values represent the mean ± SEM.

To validate the effect of endogenous Hhip, we used male heterozygous Hhip (Hhip^+/−^) mice [N.B., Adult Hhip^+/–^ mice are phenotypically indistinguishable from control littermates (Hhip^+/+^), whereas Hhip null mice (Hhip^−/−^) die after birth due to lung defects; thus Hhip^+/−^ were used in the current study^7,14^]. We examined the renal outcomes in male Hhip^+/−^ vs. Hhip^+/+^ mice undergoing 4 weeks of LDSTZ -induced diabetes from the age of 12 to 16 week-old (Fig. 6a). In the non-diabetic condition, adult Hhip^+/–^ mice were indistinguishable from Hhip^+/+^ mice including kidney weight (KW)/tibia length (TL) ratio (Fig. 6b), glomerular filtration rate (GFR) (Fig. 6c), urinary albumin/creatinine ratio (ACR) (Fig. 6d) and renal morphology (Fig. 6e, Periodic-Acid Schiff (PAS), Masson, Nox4-IHC and podocyte numbers). After 4-weeks of diabetes, diabetic Hhip^+/+^ animals had evidence of renal hypertrophy (Fig. 6b), increased GFR (Fig. 6c) and urinary ACR (Fig. 6d), and developed DN features (Fig. 6e) including glomerulosclerosis, glomerular fibrosis and podocyte loss (co-IF staining with p57 and Hhip), as well as elevated oxidative stress (Nox4-IHC). However, such renal changes were attenuated in diabetic Hhip^+/−^ mice (Fig. 6b–e) with normal urinary ACR (Fig. 6d). Systolic blood pressure (SBP) (Fig. 6f), however, remained unchanged among the different groups with or without 4-weeks of diabetes.

## Discussion

In the current study, we systematically examined renal Hhip expression in several diabetic murine models. We demonstrated that Hhip expression is significantly increased in diabetic GECs and that kidney injury is ameliorated in diabetic Hhip^+/−^ mice. Our data support the notion that augmented renal Hhip expression may play an important role in the progression of DN by promoting apoptosis and fibrosis in GECs.

While GEC injury is the hallmark of early renal injury in DN, there is a pressing need to identify novel insights into causal processes that contribute to the onset of diabetes-related glomerular endothelial injury or its progression that may help to identify potential therapeutic targets^1–3^. Here, we are focusing on Hhip, a molecule not previously considered in the development and progression of DN; we found that Hhip appears to be involved in GEC injury in diabetic murine models, a novel finding.

In the kidney, the functional role of renal Hhip expression in both developing and mature kidneys is unknown. Previously, we reported that renal *Hhip* gene is excessively expressed in the nascent glomeruli of the offspring of diabetic dams. Consequently, those elevated/trapped renal Hhip proteins, via Hhip-TGFβ1 interaction, lead to the impaired kidney development observed^20^. After birth, in normal non-diabetic states, Hhip expression is quiescent, with only a limited amount of Hhip detectable in mature GECs in line with endothelial cells Hhip-expressing property^11^, however, it is not detectable in podocytes by immunohistochemistry^20^. In contrast, in animals with diabetes, we observed that renal Hhip expression was significantly elevated in mature kidneys (Akita, *db/db* and LDSTZ-induced diabetic models), predominantly localized to GECs (confirmed by CD31 co-localization). We further confirmed the stimulatory effect of high glucose on pGL4.20/mHhip promoter activity, Hhip mRNA and protein expression in a dose- and time-dependent manner; Hhip induces mECs apoptosis and EndoMT *in vitro*. Moreover, an increased Hhip expression was also observed in mouse podocytes cultured in high glucose milieu. Consistent with our recent findings^30^, when Hhip expression is ectopically activated and/or stimulated in podocyte, Hhip could trigger caspase-3 and p53-related apoptotic processes resulting in podocyte loss and activate TGFβ1-Smad2/3 cascades and α-SMA expression to transform differentiated podocytes to undifferentiated podocyte-derived fibrotic cells^30^. Taken together, our data indicate that increased Hhip expression might directly impact diabetes-related glomerular endothelial injury.

Counterbalance between Hhip and Shh signaling appears to be important for maintaining a normal Shh gradient [distal (high) to proximal (low)] in the developing kidney^32,33^, and interruption of the Shh gradient has been shown to result in renal dysplasia/hypoplasia^32^. In the mature kidney, although Shh has been linked to renal fibrosis^34,35^, given the fact that Hhip expression is quiescent after birth, it is unclear whether high glucose-promoted *Hhip* gene expression could function dependently and/or independently via Shh/Ptc1-signaling process. Our present studies revealed that Shh expression was highly elevated in the renal cortex of diabetic models (Akita and *db/db* mice). *In vitro*, neither high glucose nor rHhip had an impact on Shh protein expression, suggesting that high glucose regulation of Hhip and TGFβ1 gene expression may take place prior to the Shh/Ptcl signaling pathway, underscoring the independence of Hhip action.

It has been well-established that hyperglycemia and Ang II increase cellular oxidative stress (reactive oxygen species, ROS) and play key roles in the pathogenesis of DN^3,36,37^. Our data showed that Ang II and H_2_O_2_ directly and dose-dependently promote Hhip expression and the TGFβ1-Smad2/3 cascades and that catalase attenuates the stimulatory effect of high glucose on Hhip protein expression. Hhip is known to play a critical role in cell apoptosis, angiogenesis and tumorigenesis^11,19,38^. While several mechanisms involving cross-talk among oxidative stress, hedgehog signaling, and TGFβ1 signaling have been associated with certain pathologic conditions such as diabetic retinopathy^39^, brain ischemia^40^, renal fibrosis^34,35^, pulmonary fibrosis^41^ and cancer-related epithelial to mesenchymal transition and metastasis^42^. Together, these observations led us to hypothesize that Hhip could interact with ROS and/or TGFβ1-signaling to result in EndoMT associated with DN-fibrosis/apoptosis.

TGFβ1 has been implicated in DN-related EndoMT transition^43,44^ and Nox4-derived ROS have a central role in TGFβ1-related EndoMT in renal fibrosis^45^. In contrast to superoxide-generating enzymes Nox1 and Nox2, Nox4 is an H_2_O_2_-generating enzyme and is highly expressed in murine endothelial cells^31^. Our studies showed elevated Nox4 IHC-staining in the glomeruli of both Akita and *db/db* mice and that the stimulatory effect of high glucose on Hhip and TGFβ1 mRNA expression in mECs could be completely abolished by GKT137831, demonstrating the involvement of Nox4. Furthermore, rHhip dose-dependently elevated ROS generation as revealed by DHE and Nox4 IF staining in mECs, and that H_2_O_2_ stimulates Hhip production, underscoring a positive feedback loop of Hhip and ROS, probably mediated by Nox4. Consequently, rHhip triggers mECs apoptosis (increases in TUNEL-positive cells and cleaved caspase-3) and fibrosis (increases in Fn1, α-SMA, and p27 expression).

Our studies also revealed that renal Hhip and TGFβ1 had similar expression patterns in diabetic kidneys; *in vitro*, both Hhip and TGFβ1 genes shared a similar time-course responding to high glucose stimulation in mECs, and rHhip stimulated TGFβ1 transcription and TGFβ1-Smad2/3 cascade signaling, together, underscoring a functional interaction of Hhip and TGFβ1. Indeed, Hhip siRNA attenuated the effect of high glucose on TGFβ1 production. Blocking TGFβ receptors (TGFβ1RI by SB431532 or TGFβ1RII by siRNA) completely attenuated the action of rHhip or high glucose on TGFβ1 expression. At the present, we do not understand how Hhip interacts with TGFβ1. One possibility might be that Hhip acts upstream of TGFβ1 signaling, targeting TGFβ1 gene transcription and translation. This is supported by our data that rHhip stimulates TGFβ1 promoter activity, protein expression and TGFβ1-Smad2/3 cascade signal. Furthermore, our data showed that rTGFβ1 fails to impact Hhip promoter and protein expression, but it directly promotes fibrotic gene expression (Shh, α-SMA, and p27). Together, these data strongly support the presence of the axis of Hhip-TGFβ1-Shh and its action on DN-related EndoMT.

To further support the role of renal Hhip in DN progression, we extended our investigation in LDSTZ-induced diabetic heterozygous Hhip^+/–^ mice. Our data revealed that adult non-diabetic male Hhip^+/−^ mice exhibit grossly normal renal morphology, similar to control Hhip^+/+^ mice. Diabetic Hhip^+/+^ mice had a significant increase in urinary ACR in a time-dependent manner and exhibited features that were consistent with DN progression (renal hypertrophy, increased GFR, glomerulosclerosis/fibrosis and podocyte loss). In contrast, diabetic Hhip^+/−^ mice had normal urinary ACR with less pronounced renal morphologic and/or functional changes. Such data indicate that with a lower renal Hhip expression, the kidney is protected from DN development. Consistent with the notion that GEC injury may lead to podocyte damage, and that podocyte loss further exacerbates GEC injury, forming a vicious cycle ultimately leading to DN that has been gaining attention^1–3^, our data indicate that augmented Hhip gene expression is associated with podocyte transition from a normal morphology to an apoptotic and/or fibrotic-like phenotype, and are further supported by the finding that human HHIP is upregulated in glomerular cells, in patients with focal segmental glomerulosclerosis^30^. However, GECs specific gain- and/or loss-of-Hhip function/expression murine models would be needed to circumvent the potential pitfall of whole body Hhip-deficient murine model in the future. Taken together, the present data support the concept that Hhip plays a role in diabetic nephropathy; further studies will be needed to elucidate the mechanism(s) of cross-talk between GECs and/or podocyte-derived Hhip functions on EndoMT. Finally, blood pressure remained unchanged, consistent with other studies in a LDSTZ model^46^.

In summary, our findings document that Hhip expression is associated with nephropathy development in diabetes and that hyperglycemia-induced renal Hhip expression may mediate glomerular endothelial fibrosis and apoptosis in diabetes.

## Methods

### Animal Models

Adult male wild-type (WT, C57BL/6 J), heterozygous Akita mice with a mutated *insulin2* gene (C57BL/6-Ins2^Akita^/J), heterozygous *db/m* and homozygous *db/db* mice (C57BLKS) were purchased from Jackson Laboratories (Bar Harbor, ME: http://jaxmice-jax.org). All animals were fed standard mouse chow and water *ad libitum*. These mice were studied from 12 to 20 weeks of age, as reported elsewhere^22–24,26,27^.

Male heterozygous Hhip (Hhip^+/−^) mice and control littermates (Hhip^+/+^) (Jackson Laboratories, Hhip^*tm1Amc*^/J; mixed background) were used^7,14^. The low-dose streptozotocin (STZ, Sigma-Aldrich Canada Ltd., Oakville, ON, Canada) (LDSTZ) model^47^, as recommended by the NIH Animal Models of Diabetic Complications Consortium (http://www.diacomp.org/), was performed in Hhip^+/+^ and Hhip^+/−^ mice at 12 weeks of age (i.e., intraperitoneal injections of STZ at ~45–50 mg per kg body weight (BW) daily for 5 consecutive days). Mice were then euthanized at the age of 16 weeks after a 4-week experimental period. Blood glucose levels were measured with an Accu-Chek Performa glucose meter (Roche Diagnostics, Laval, QC, Canada) in the morning after a 4-hour fast^24,25^. Mice with blood glucose levels below 16 mmol/l (measured 72 hours following the last STZ injection) were excluded from our analyses.

Systolic blood pressure (SBP) was monitored by the tail-cuff method with a BP-2000 Blood Pressure Analysis System (Visitech Systems Inc., Apex, NC), as reported elsewhere^23,48,49^. The non-diabetic and LDSTZ-induced diabetic mice (N = 5–7 mice/group) were acclimated to SBP measurement to minimize stress (training period of 5 days during 3^rd^ week of STZ-induction), and then actual SBP was measured for 5 consecutive days during the 4^th^ week of the experiment (the average SBP of 5 days of measurements were reported). We counted podocyte numbers per glomerulus that were positive for p57 (a marker for podocytes; 5–6 mice/group; 30–40 glomeruli/animal) and compared the results among the groups in a blinded fashion^30^.

All animal protocols were carried out in strict accordance with the recommendations in the NIH Guide for the Care and Use of Laboratory Animals and followed the Principles of Laboratory Animal Care [National Institutes of Health (NIH) publication no. 85-23, revised 1985: http://grants1.nih.gov/grants/olaw/references/phspol.htm]. Animal care and procedures were approved by the Animal Care Committee from the Centre de recherche du centre hospitalier de l’Université de Montréal (CRCHUM).

### Cell Lines and Promoter Analysis

The murine SVEC4-10 endothelial cell line (mECs) (ATCC, CRL-2181) was a kind gift from Dr. Hongyu Luo (CRCHUM, Montreal, QC, Canada). The immortalized mouse podocyte cell line (mPODs) obtained from Dr. Stuart J. Shankland (University of Washington, Seattle, WA, USA) is highly proliferative when cultured under permissive conditions and has been well characterized^28–30^.

We cloned the mouse Hhip promoter (pGL4.20/mHhip, N-1542/N+9, NC_000074.6) and rat TGFβ1 promoter (pGL4.20/rTGFβ1, N-1016/+143, NM_021578.2) by PCR. Both pGL4.20/mHhip and pGL4.20/rTGFβ1 promoter activities in mECs under normal (5 mM D-glucose) and high D-glucose (25 mM D-glucose) ± recombinant Hhip (rHhip) (R&D Systems, Inc.) or rTGFβ1 (R&D Systems, Inc.) conditions were analyzed by luciferase assay.

### Immunohistochemical Studies and Reagents

Western blotting (WB), immunohistochemistry (IHC), immunofluorescence (IF) and dihydroethidium (DHE) and terminal deoxynucleotidyl transferase dUTP nick end labeling (TUNEL) staining were performed as described elsewhere^20,23,30,49^. The antibodies used for IHC and IF included the following: anti-Hhip (monoclonal clone 5D11), α-smooth muscle actin (α-SMA) and β-actin antibodies from Sigma-Aldrich Canada; Shh, TGFβ receptor II (TGFβRII), CD31, synaptopodin (Synpo) (P-19) and p57 (H-91) antibodies from Santa Cruz Biotechnology (Santa Cruz, CA, USA); p27^Kip1^ antibody from BD Biosciences (San Jose, CA, USA); cleaved caspase-3 (Asp175) and caspase-3 antibodies from Cell Signaling (Danvers, MA, USA); anti-TGFβ1 antibody from R&D Systems, Inc. (Burlington, ON, Canada); Phospho-Smad2 (Ser465/467)/Smad3 (Ser423/425) antibody (New England Biolabs, Whitby, ON, Canada); Smad2/3 antibody (Cedarlane-Millipore, Burlington, ON, Canada); and anti-NADPH oxidase 4 (Nox4) antibody (Abcam, Cambridge, MA, USA). GKT137831 (dual inhibitor of both Nox1 and Nox4) was procured from Cayman Chemical (Ann Arbor, MI, USA).

### Renal Morphology, Glomerular Filtration Rate, Urinary Albumin/Creatinine Ratio

Kidney sections were stained with Periodic-Acid Schiff (PAS) and Masson’s trichrome to reveal renal morphologic changes^23,48^. The changes of DN features—glomerulosclerosis (based on PAS images, scale from 0 to 4) and glomerular fibrosis (based on Masson staining) were scored with the scorer blinded to the group^23,48^. Relative staining was quantified with NIH Image J software (Bethesda, MD, USA). The images (N = 6~10 per animal, 6–11 mice/group) were analyzed and quantitated in a blinded fashion^23,48^. Glomerular filtration rate (GFR) was measured in conscious mice by the fluorescein isothiocyanate-inulin method as reported previously^23,49^, as recommended by the Diabetic Complications Consortium (http://www.diacomp.org/). Urine samples, collected from mice individually housed in metabolic cages, were assayed for albumin/creatinine ratio (ACR) (Albuwell and Creatinine Companion, Exocell Inc., Philadelphia, PA, USA)^23,49^.

### Statistical Analysis

For animal studies, groups of 6 to 12 mice were used. *In vitro*, three to four separate experiments were performed for each protocol. All values represent mean ± SEM. Statistical significance between the experimental groups was analyzed by Student’s *t*-test or 1-way ANOVA, followed by the Bonferroni test using Prism 5.0 software (GraphPad, San Diego, CA, USA). A probability level of *p* < 0.05 was considered to be statistically significant (*p ≤ 0.05; **p ≤ 0.01; ***p ≤ 0.001; NS, non-significant).

## Electronic supplementary material

Supplementary Information
