# Supplementary material for: Hedgehog Interacting Protein Promotes Fibrosis and Apoptosis in Glomerular Endothelial Cells in Murine Diabetes
Source: Sci Rep. 2018 Apr 13;8:5958. doi: 10.1038/s41598-018-24220-6 (PMC5899163; doi:10.1038/s41598-018-24220-6)
Supplement: Supplementary file 1 — Supplementary Information [file 41598_2018_24220_MOESM1_ESM.pdf]

Hedgehog Interacting Protein Promotes Fibrosis and Apoptosis in Glomerular Endothelial Cells in Murine Diabetes

Xin-Ping Zhao[1], Shiao-Ying Chang[1], Min-Chun Liao[1], Chao-Sheng Lo[1], Isabelle Chenier[1], Hongyu Luo[1], Jean-Louis Chiasson[1], Julie R. Ingelfinger [2], John S.D. Chan[1] and Shao-Ling Zhang [1§]

[1]Université de Montréal
Department of Medicine
Centre de recherche du Centre hospitalier de l'Université de Montréal (CRCHUM)
Tour Viger, 900 rue Saint-Denis, Montréal, QC, Canada H2X 0A9

[2] Harvard Medical School
Pediatric Nephrology Unit
Massachusetts General Hospital
55 Fruit Street, Boston, MA 02114-3117, USA

The authors declare that there is no potential conflict of interest relevant to this article.

[§] To whom correspondence should be addressed: Shao-Ling Zhang, Ph.D.

Tel: (514) 890-8000 ext. 15633
Fax: (514) 412-7204
Email: shao.ling.zhang@umontreal.ca

Short Title: Hedgehog interacting protein (Hhip) in diabetic nephropathy
Key words: Hhip gene expression, diabetic nephropathy, glomerular endothelial cells (GECs)
Word Count: 14 words; Abstract: 182 words; Text: 2683

Supplemental Figure 1

Fig. S1a

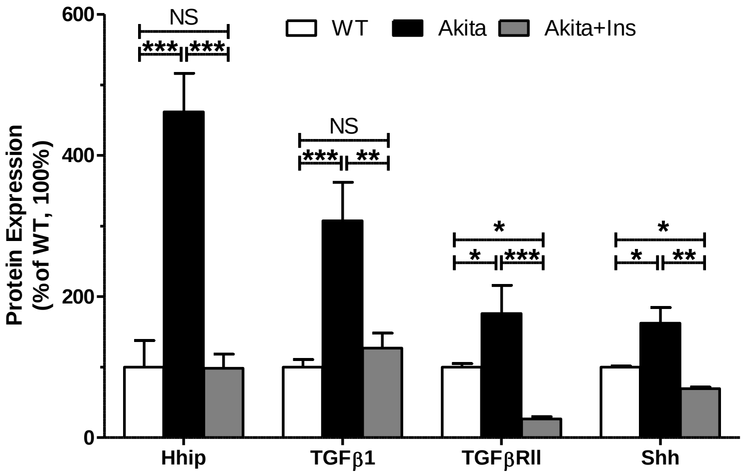

Fig. S1b

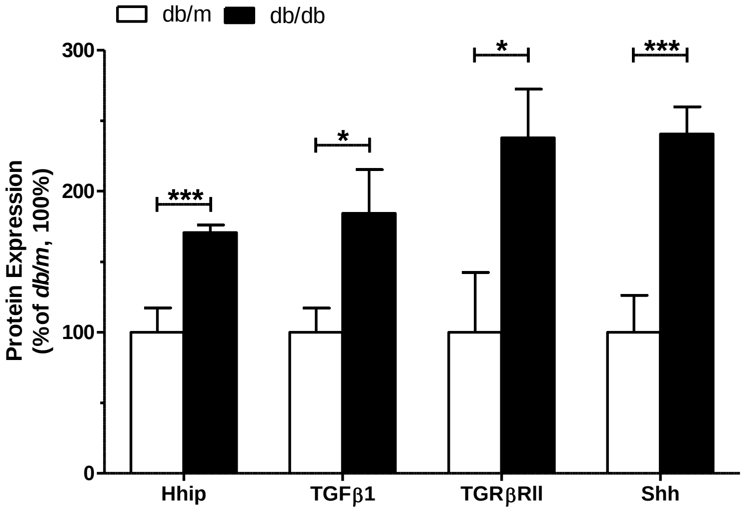

Supplemental Figure 2

Fig. S2a

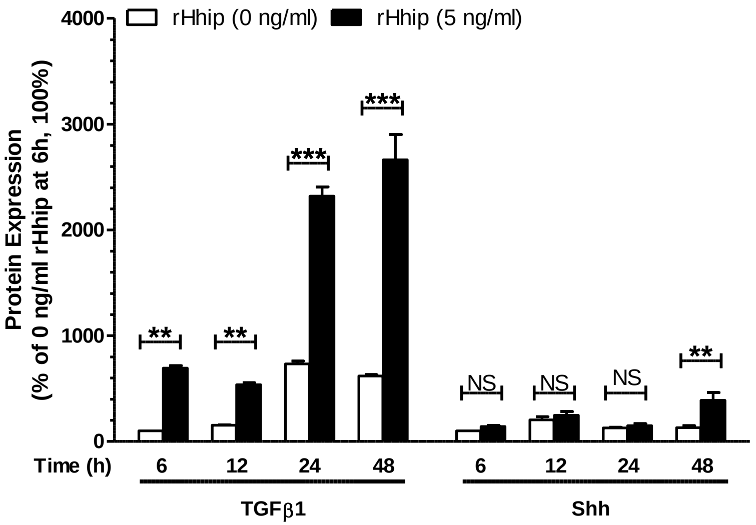

Fig. S2b

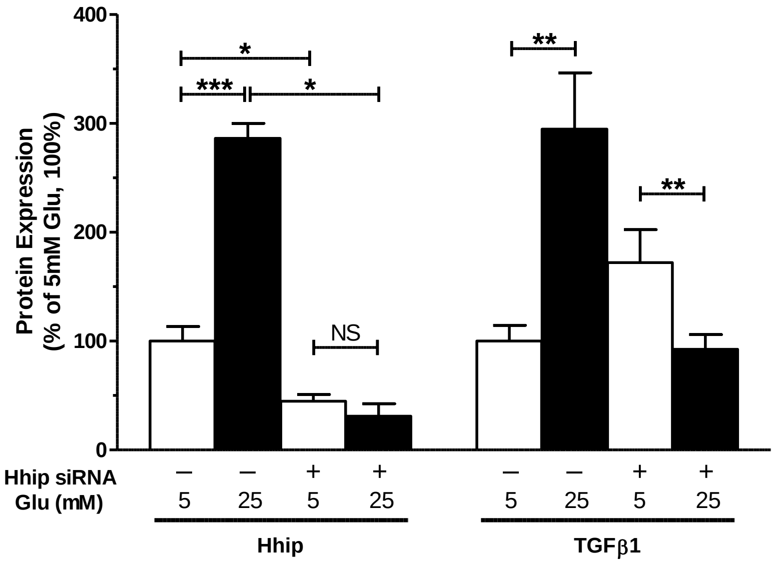

Fig. S2c

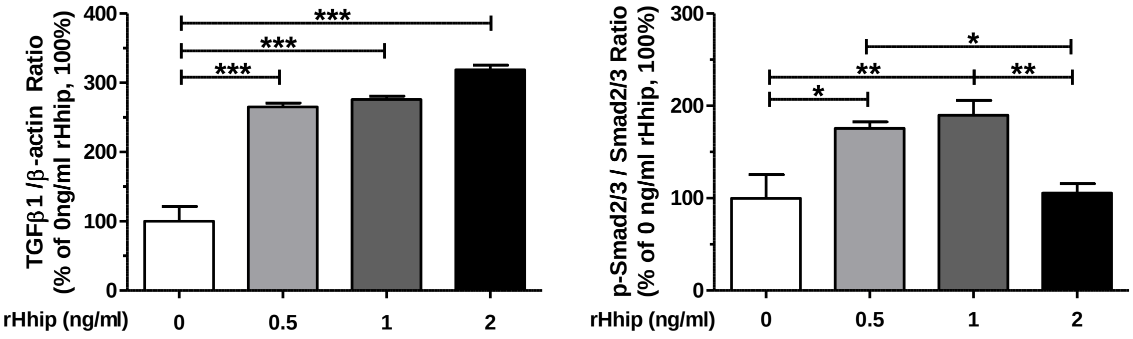

Supplemental Figure 3

Fig. S3

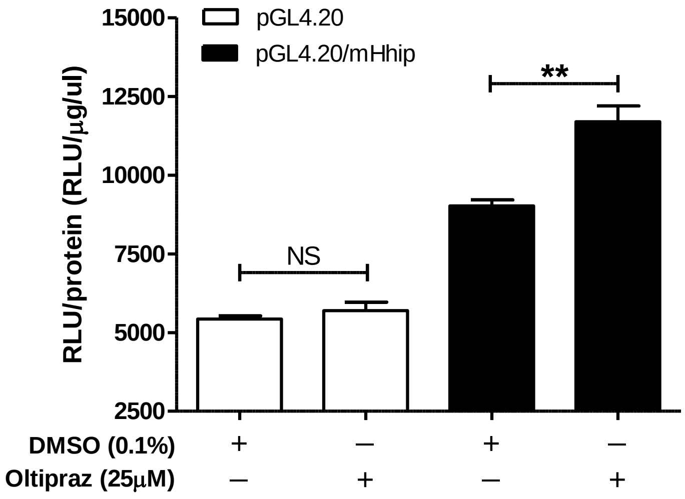

SUPPLEMENTAL FIGURE LEGEND

S1. Semi-quantification of WB analysis in Akita (S1a--Figure 1b) and *db/db* mice (S1b--Figure 1e). *$P \leq 0.05$; **$P \leq 0.01$; ***$P \leq 0.001$; NS, non-significant; Values represent the mean ± SEM.

S2. Semi-quantification of WB analysis (S2a-S2c). S2a (Figure 5a); S2b (Figure 5b) and S2c (Figure 5d), *$P \leq 0.05$; **$P \leq 0.01$; ***$P \leq 0.001$; NS, non-significant. Values represent the mean ± SEM.

S3. Oltipraz (an Nrf2 activator, 25µM) stimulates pGL4.20/mHhip promoter activity analyzed by luciferase assay, **$P \leq 0.01$; ***$P \leq 0.001$; NS, non-significant vs. mECs transfected with pGL4.20 (1µg/ml) (100%); Values represent the mean ± SEM.
